# Supplementary material for: Anp32a Promotes Neuronal Regeneration after Spinal Cord Injury of Zebrafish Embryos
Source: Int J Mol Sci. 2022 Dec 14;23(24):15921. doi: 10.3390/ijms232415921 (PMC9786895; doi:10.3390/ijms232415921)
Supplement: Supplementary file 1 [file ijms-23-15921-s001.zip › ijms-1957451-supplementary.pdf]

Table S1. Morphological phenotypes and swimming capacity of SCI-embryos injected with different materials as indicated.

Materials	Amount	No. of survival embryos among injected eggs	No. of wild-type like phenotype	Average of swimming distance (cm)
Uninjured embryo	N/A	36/36	36/36	5.76681
non-injection	N/A	48/53	47/48	3.59689
anp32a-MO	1 ng	48/63	46/48	3.57431
anp32a-MO	1.5 ng	45/62	40/45	3.43722
anp32a-MO	2 ng	74/102	63/74	1.85472
anp32a-MO	4 ng	11/47	3/11	0
non-injection	N/A	42/44	42/42	3.63248
anp32a RNA	200 pg	54/64	39/54	5.72380
anp32a RNA	400 pg	43/82	11/43	3.58293
Wobble anp32a flag RNA	200 pg	38/51	29/38	5.43829
Wobble anp32a flag RNA	400 pg	33/84	9/33	3.11833
anp32a-MO+ *Wobble anp32a flag* RNA	2 ng + 200 pg	45/67	38/45	4.67383

All embryos except uninjured embryo group were SCI at 48 hpf and performed swimming capacity assay at 24 hpi
